# Examining the relationship between ecological anxiety and pro-environmental behavior: personal and collective actions

**DOI:** 10.3389/fpsyg.2025.1505564

**Published:** 2025-08-13

**Authors:** Gal Hagit Carasso Romano, Rotem Sippori, Sharon Soroker

**Affiliations:** ^1^Faculty of Social Sciences & Humanities, Tel-Hai College, Tel Hai, Israel; ^2^Academic Center of Law and Science, Hod Hasharon, Israel

**Keywords:** ecological anxiety, environmental psychology, environmental awareness, collective pro-environmental action, personal pro-environmental action, climate crisis

## Abstract

Ecological anxiety—defined as anxiety related to environmental degradation and climate change—has become increasingly prevalent, particularly among individuals who are environmentally conscious. This study investigated the correlation between ecological anxiety and both personal and collective pro-environmental behavior among 224 participants, predominantly members of environmental groups with inherent environmental concern. The study aimed to clarify the extent to which ecological anxiety motivates different forms of environmental behavior, and whether personal and collective actions serve similar psychological functions. Using Pearson’s correlation and regression analysis, the research identified significant positive correlations between ecological anxiety and both forms of environmental behavior, with a notably stronger correlation for collective behavior. The study also examined whether environmental actions serve as coping mechanisms that provide individuals with a sense of control over uncontrollable environmental situations, or alternatively, whether these actions could exacerbate anxiety among those actively engaged in addressing environmental challenges. These findings are particularly relevant for mental health professionals, environmental educators, and policymakers, as they emphasize the need to consider both personal and collective dimensions of environmental behavior. Integrating this perspective into educational programs and policy design may help transform ecological anxiety into a constructive force for environmental engagement and resilience.

## Introduction

### Background and definitions

The global environmental crisis, particularly the phenomenon of climate change, presents one of the most critical challenges of our time. Decades of research, dating back to the 1970s, have established the significant human contributions to environmental degradation and the severe, long-term implications of climate change for the planet and its inhabitants (Wright, 1970; Ripple et al., 2020). As awareness of these issues has grown, so too their psychological impact, giving rise to a condition known as ecological anxiety (Pihkala, 2020a). The American Psychological Association (APA) defines ecological anxiety as a persistent fear triggered by environmental threats such as climate change, biodiversity loss, and pollution. This form of anxiety is characterized by feelings of loss, helplessness, and frustration concerning the deteriorating state of the environment (Coffey et al., 2021).

### Prevalence and demographic variations

Recent studies indicate that ecological anxiety is highly prevalent, with approximately 70–80% of the adult population experiencing some level of this anxiety (Ágoston et al., 2022a; Lee, 2023; Brophy et al., 2023).

Climate change, being a visible and critical aspect of the broader environmental crisis, is a focal point for ecological anxiety. Individuals directly affected by climate change are more likely to develop this anxiety (Ágoston et al., 2022a; Stanley et al., 2021); yet, even those not directly impacted can experience significant distress through media exposure and increasing global awareness (Loll et al., 2023). Notably, demographic factors such as gender, age, and living environment influence the prevalence of ecological anxiety, with women, younger individuals, and those in rural areas more prone to higher anxiety levels than their respective opposites (Coffey et al., 2021; Gifford and Gifford, 2016; Clayton and Karazsia, 2020).

### Cognitive and emotional aspects

The unique characteristics of the environmental crisis, including its global scale, evolving nature, and lack of straightforward solutions, contribute to a fertile ground for anxiety. Warnings of reaching a point of no return and the unpredictability of the crisis’s effects amplify feelings of uncontrollability and uncertainty, thereby intensifying anxiety (Ojala et al., 2021). From a psychological perspective, ecological anxiety involves both cognitive assessments of environmental threats and affective responses such as fear, grief, and despair. These emotional reactions may influence perceptions of agency, urgency, and responsibility toward environmental issues.

### Existential dimensions and implications

Ecological anxiety often encompasses existential and apocalyptic elements, as individuals grapple with fundamental questions of life and death and the anticipated widespread impact on human life due to migration (Bonneux and Van Praag, 2024), human suffering, and potential global conflicts stemming from the climate crisis (Pihkala, 2020a).

### Need for interventions

Understanding the interplay between various environmental actions and ecological anxiety is crucial for developing therapeutic interventions and supporting those affected by this condition. Environmental Identity Theory (Clayton, 2003) offers an important theoretical lens, suggesting that individuals who see nature as part of their self-concept are more likely to experience ecological anxiety but also more inclined to take environmental action.

This framework supports the need to explore how different forms of engagement may either alleviate or intensify eco-anxiety and can inform psychological and educational responses.

### Ecological anxiety and pro-environmental action

Building on Environmental Identity Theory and recent psychological models, a significant aspect of the discourse on ecological anxiety pertains to its relationship with pro-environmental behavior. Although ecological anxiety is not a prerequisite for engaging in pro-environmental actions, it is a strong predictor of such behavior (Jain and Jain, 2022). Engaging in environmental activities provides individuals with a sense of control that can mitigate distress, serving as a therapeutic channel for converting feelings of helplessness into empowerment and fostering hope (Nairn, 2019; Ojala, 2023; Coppola, 2021). Environmental education also plays an important role in supporting and building emotional resilience among those coping with ecological anxiety (Pihkala, 2020b). Additionally, the role of mental health professionals is highlighted in treating ecological anxiety through interventions aimed at building resilience through pro-environmental actions and enhancing connections with nature (Baudon and Jachens, 2021).

Conversely, direct engagement with environmental crises can heighten concern and generate negative psychological effects in personal lives (Ojala, 2013; Clarke, 2006; Venhoeven et al., 2013), especially when the stressor is uncontrollable (Clarke, 2006).

Another layer in the complex relationship between ecological anxiety and pro-environmental action is the distinction between individual and collective pro-environmental behavior (Stanley et al., 2021). Personal pro-environmental behavior involves everyday actions that impact environmental aspects and climate change, such as reducing meat consumption, wearing second-hand clothes, collecting household water for watering the garden, reducing personal consumption, and changing car usage habits (Brick and Lewis, 2016). Collective pro-environmental behavior refers to actions taken by individuals in a group or organization to influence the environment in a structured manner, such as participating in climate marches, funding or donating to organizations, voting or encouraging voting for parties with declared environmental policies, and writing letters to companies producing polluting products (Sguin et al., 1998).

Each of these behaviors can have a bidirectional impact. Personal pro-environmental behavior may reduce anxiety because it provides a sense of control, or it may increase anxiety due to the heightened focus on the environment and the impact of our actions on it and the climate crisis (Stanley et al., 2021). Similarly, the collective discourse on environmental crises and heightened responsibility toward others that accompanies collective action may increase anxiety. In contrast, the sense of partnership and the ability to share feelings with peers may mitigate anxiety (Nairn, 2019).

However, while previous studies have demonstrated an association between ecological anxiety and pro-environmental behavior, their findings regarding the nature of this relationship have been inconsistent. Moreover, although some studies have differentiated between personal and collective forms of pro-environmental behavior, few have systematically examined how each uniquely relates to ecological anxiety, or which of the two contributes more significantly. The present study aims to address this gap by exploring the distinct associations between ecological anxiety and both personal and collective pro-environmental behaviors. By doing so, it seeks to provide a more nuanced understanding of how different types of environmental engagement may interact with ecological anxiety.

## Methods

### Research procedure and sampling

The research was conducted in the summer of 2023 with participants aged 19–80, recruited through a Qualtrics link distributed on social media in environmental groups. To create a diverse sample of respondents in terms of age and types of environmental engagement, the survey was distributed to several groups focused on Personal Pro-Environmental Behavior (such as “Zero Waste” Facebook group and The “Green Collar” Facebook group focused on environmental career opportunities) as well as those who engage in Collective Pro-Environmental Behavior (such as “Extinction Rebellion Israel” and environmental leadership WhatsApp groups). Due to their affiliation with these groups, respondents were expected to have a high level of environmental awareness and to likely experience some degree of anxiety. Participation was voluntary.

To ensure alignment with the study’s focus on ecological anxiety among individuals who recognize climate change as anthropogenic, participants were first asked a screening question (adapted from Stanley et al., 2021): ‘What best describes your thoughts on climate change? I believe climate change is happening, and I think humans are causing it’. Responses were rated on a 5-point Likert scale (1 = strongly agree, 5 = strongly disagree). Participants who selected 4 or 5 were excluded from the study, as the emotional experience of individuals who deny climate change and/or its anthropogenic origins is assumed to differ from those who recognize human-induced climate change (Myers et al., 2012). Two respondents did not pass the threshold question, and the Qualtrics system prevented access to the full questionnaire.

Demographic Information:

A total of 224 participants completed the full survey.

Gender: 153 women (68.3%) and 71 men (31.7%).
Age: Participants ranged in age from 18 to 80 years (*M* = 37, *SD* = 13.97).
Geographic Location: 57.4% lived in central Israel, 34.5% in the north, and 8.1% in the south.
Living Environment: 61.9% resided in urban areas, while the rest lived in various rural settings: Kibbutzim (11.7%), Moshavim (13.5%), communal settlements (9.4%), villages (0.9%), and Moshavot (2.7%).

### Research questionnaires and variable measurement

Three well-known questionnaires from the research literature whose reliability has been demonstrated were translated into Hebrew and combined into one survey (Ágoston et al., 2022a; Brick and Lewis, 2016; Sguin et al., 1998; Likert scale 1–5). Their internal reliability was examined using Cronbach’s alpha and McDonalds’ omega and is presented in Table 1.

**Table 1:** Research variables values according to questionnaire results (*n* = 224) Likert Scale 1–5.

Variable	Range	Mean ± SD	Cronbach’s alpha	Upper CI	Lower CI	Mcdonald’s W	Upper CI	Lower CI
Personal pro-environmental behavior	2–5	3.25 ± 0.52	0.72	0.767	0.666	0.714	0.769	0.659
Collective pro-environmental behavior	1–5	2.32 ± 0.85	0.904	0.921	0.884	0.905	0.923	0.886
Overall pro-environmental behavior	1.4–4.3	2.95 ± 0.55	0.806					
Ecological anxiety	1.59–5	3.13 ± 0.69	0.84	0.84	0.76	0.814	0.852	0.84

*Questionnaire 1 – Personal Pro-Environmental Behavior* (Supplementary Appendix 1). This questionnaire examines 15 individual behaviors that impact the environment, such as the frequency of using reusable bags and recycling household waste. The questionnaire was developed and applied by Brick and Lewis (2016).

*Questionnaire 2 – Activism and Collective Pro-Environmental Behavior* (Supplementary Appendix 2). This questionnaire includes 7 questions based on interviews with activists, addressing participation in environmental events, financial contributions to organizations, signing petitions, participating in protests, and influencing policy. The questionnaire was developed and applied by Sguin et al. (1998).

*Questionnaire 3 – Ecological Anxiety* (Supplementary Appendix 3). This questionnaire includes 22 questions assessing levels of ecological anxiety, such as anxiety stemming from environmental pollution, awareness of natural disasters, and climate change. The questionnaire was taken from a study by Ágoston et al. (2022b).

Respondents also noted demographic information including age, gender, and place of residence.

### Statistical analysis

Data analysis was conducted using JASP (0.14.1.0). The internal reliability of the questionnaires was examined using standard measures for assessing internal consistency of scales; Cronbach’s alpha (Cronbach, 1951) and McDonalds’ omega (Table 1). Statistical relationships between anxiety levels and personal and collective pro-environmental behavior were examined using Pearson’s correlation, appropriate for evaluating linear associations between continuous variables (Chapman, 2017). The influence of personal pro-environmental behavior and ecological anxiety on activism and collective pro-environmental behavior were examined using linear regression and ANOVA tests, which are suitable for modeling predictive relationships and comparing group means, respectively (Cohen et al., 2003).

A power analysis was conducted prior to the study to estimate the necessary number of respondents required to achieve statistically significant results (Supplementary Appendix 4) (Schäfer and Schwarz, 2019; Sternberg et al., 2020; Shabat et al., 2021).

## Results

Of 226 respondents, 224 passed the screening question acknowledging the existence of climate change and attributing it to human actions. Among them, 47% had an average response greater than 3 on both questionnaires measuring pro-environmental behavior (on a scale of 1–5), indicating that they engage in various actions to mitigate the crisis, both individually and collectively.

### Personal and collective pro-environmental behavior

A Pearson correlation analysis revealed a significant positive relationship between personal and collective pro-environmental behavior (*r* = 0.568, *p* < 0. 001). Additionally, the mean score for personal pro-environmental behavior was higher than that for collective pro-environmental behavior, suggesting a greater tendency for individual actions compared to collective actions (Table 1).

Table 1 presents the values of the research variables and the reliability of the questionnaires as found in our study. The response options were on a Likert scale ranging from 1 (strongly disagree) to 5 (strongly agree), and the mean represents the average responses for each questionnaire separately.

### Anxiety and pro-environmental behavior

The relationship between ecological anxiety and pro-environmental behavior was examined using Pearson correlation tests. Separate analyses were conducted for each type of pro-environmental behavior: personal and collective. For personal pro-environmental behavior, a weak but significant positive correlation was observed (*r* = 0.30, *p* < 0.001). In contrast, for collective pro-environmental behavior, a moderate positive and significant correlation was found (*r* = 0.51, *p* < 0.001) (Table 2). These results suggest that ecologica anxiety is more strongly associated with collective pro-environmental behavior than with personal behavior.

**Table 2:** Pearson correlation coefficients.

Variable	Ecological anxiety	Personal pro-env behavior	Collective pro-env behavior
Ecological anxiety	–		
Personal pro-env behavior	0.30**	–	
Collective pro-env behavior	0.51**	0.57**	–

### Predicting collective pro-environmental behavior

To examine the predictors of collective pro-environmental behavior, a linear regression analysis was conducted with personal pro-environmental behavior and ecological anxiety as predictors. The model was significant, explaining a substantial portion of the variance in collective pro-environmental behavior [adjusted R^2^ = 0.674, *F*(2,223) = 232.23, *p* < 0.001].

Both personal pro-environmental behavior (*t* = 4.079, *p* < 0.001) and ecological anxiety (*t* = 19.683, *p* < 0.001) significantly contributed to the model.

Ecological anxiety emerged as the stronger predictor, with a larger part correlation (0.749), indicating that it uniquely accounts for a considerable portion of the variance in collective behavior beyond the contribution of personal behavior. The partial correlation (0.797) further shows that the relationship between ecological anxiety and collective behavior remains strong even when controlling for personal behavior. In contrast, the part and partial correlations for personal behavior (0.155 and 0.264, respectively) reflect a smaller, yet significant, contribution. (Table 3) These results suggest that ecological anxiety is a key independent predictor of collective pro-environmental behavior, while personal behavior plays a complementary role.

**Table 3:** Predicting collective pro-environmental behavior.

Predictor	*t*	*p*
(Intercept)	4.349	<0.001
Personal pro-env behavior	4.079	<0.001
Ecological anxiety	19.683	<0.001

### Additional analysis

The correlation between age and environmental behavior was also examined using a Pearson test. Age was found positively correlated with personal pro-environmental behavior (*r* = 0.16, *p* = 0.019) and negatively correlated with ecological anxiety (*r* = −0.205, *p* < 0.01) and not significantly associated with activism and collective pro-environmental behavior (*r* = −0.127, *p* = 0.003).

## Discussion and conclusions

The results of the study indicate a positive relationship between ecological anxiety and pro-environmental actions, both individual and collective. These findings align with Ojala’s work, which suggests that problem-focused coping, i.e., active engagement by individuals facing the climate crisis, is associated with climate and ecological anxiety (Ojala et al., 2021; Ojala, 2023; 2013). This relationship may arise from two alternative trends. First, engaging in environmental actions might intensify anxiety. This view is supported by Clarke, who argues that although action-based coping strategies are generally effective, they are insufficient when the problem is large and impossible to tackle alone, leading to lower well-being (Clarke, 2006). This explanation is germane to the unique aspects of the climate crisis, including uncertainty about its impact and the relationship between it and existential anxiety, both of which amplify anxiety and the feeling that personal habit changes and collective activism do not make a difference when the situation is so critical.

Alternatively, the correlation between anxiety and pro-environmental behavior may suggest that individuals more anxious about the environment are more proactive in mitigating the crisis; they adopt a psychological coping mechanism in the face of significant global challenges. It is also possible that certain personality traits, such as high conscientiousness or prosocial tendencies, may simultaneously drive both higher ecological anxiety and increased environmental engagement, as suggested by Clayton and Karazsia (2020). Moreover, both personal and collective actions may serve as coping mechanisms for individuals experiencing ecological anxiety, offering a sense of control and purpose amid environmental uncertainty. External motivators, such as social norms within environmental groups, could reinforce both anxiety and action (Fielding and Hornsey, 2016). Engaging a broad spectrum of individuals, including those outside traditional activist circles, underscores the inclusive potential of collective action (Schwartz et al., 2023). Engaging in personal or collective environmental activism can serve to achieve a sense of control in an uncontrollable situation (Jain and Jain, 2022) and environmental education, motivation to act, and connection with nature are beneficial in cases of ecological anxiety (Pihkala, 2020b; Baudon and Jachens, 2021). Furthermore, findings that climate anxiety (a significant component of ecological anxiety) is a predictor of collective pro-environmental behavior support this alternative, highlighting the latter’s potential as a therapeutic channel for anxiety (Schwartz et al., 2023; Innocenti et al., 2023).

### Collective vs. personal pro-environmental action

The survey results demonstrate that respondents are more involved in changing personal habits for the environment than in collective actions to change the public agenda, even though collective actions have a broader impact and hence may offer a greater sense of influence and agency for those experiencing ecological anxiety. This explanation aligns with the stronger correlation found between collective pro-environmental action and ecological anxiety compared to individual action.

Several reasons can explain the difference in correlation between ecological anxiety and collective versus personal action:

- *Scale of Impact*: Since collective actions typically address larger systemic issues, individuals may feel that although personal actions are essential, they do not have the widespread impact of collective action (Kim et al., 2024). As a result, those who are more anxious may believe that large-scale changes are necessary and thus turn to collective action.
- *Sense of Influence*: Belonging to a large group can give individuals the feeling that their actions have more significant impact (Fritsche and Masson, 2021), which may attract those with heightened ecological anxiety.
- *Social Support and Shared Concern*: Collective action provides individuals with a platform to express their concerns and find comfort in a shared understanding of the issues. The communal nature of such actions may act as a psychological buffer against feelings of despair or helplessness (Dietz et al., 1998). Conversely, collective activity, itself, may increase anxiety, as the group discussion and sense that the climate crisis is complex and unsolvable can exacerbate distress. This suggests that beyond their practical impact, collective actions may fulfill psychological needs for community belonging and shared responsibility, potentially buffering individuals from the isolating effects of ecological anxiety.

Age was found to be positively correlated with personal pro-environmental behavior, suggesting that as individuals age, they place a greater importance on being environmentally active in their personal lives. Conversely, age was found to be negatively correlated with ecological anxiety, indicating that younger individuals, who tend to experience higher levels of ecological anxiety, may be less active personally. This could be because as people age, they often have more opportunities to engage in personal environmental activities due to increased financial stability, allowing for investments in sustainable technologies and products; more control over household decisions, like choosing energy-efficient appliances; and greater time availability for activities such as gardening, recycling, or volunteering for environmental causes, thus enabling them to turn their environmental concerns into actionable behaviors more effectively.

These findings also underscore the potential role of well-structured environmental policies in transforming ecological anxiety into constructive engagement, especially when designed to promote collective efficacy and inclusion.

### Policy implications

The study’s findings highlight the importance of fostering and supporting collective environmental initiatives as a means to address both environmental challenges and the psychological distress they may provoke. These findings suggest that environmental policies and educational strategies should be tailored to different age groups to maximize their effectiveness and inclusivity. Policies encouraging community-based environmental projects, supporting environmental organizations, and promoting public involvement in environmental decision-making can leverage the connection between ecological anxiety and activism for positive change (Nairn, 2019; Ojala, 2023; Coppola, 2021). Specifically, Coppola (2021) conducted interviews illustrating how involvement in environmental organizations mitigates ecological anxiety among participants by fostering a sense of community and effectiveness. This tailored approach can ensure that interventions meet the unique needs of various demographics, enhancing engagement and reducing ecological anxiety across age groups. However, in light of the findings here that environmental anxiety is correlated with collective pro-environmental activity, it is essential to consider the possibility that excessive focus on environmental issues might increase anxiety (Ojala, 2013; Clarke, 2006; Venhoeven et al., 2013), necessitating the integration of environmental activities with appropriate education and public awareness (Pihkala, 2020b; Baudon and Jachens, 2021).

### Limitations and future research

#### Methodological design

Ecological anxiety might motivate action, or alternatively, environmental engagement may increase anxiety through heightened awareness of the crisis. However, due to the cross-sectional nature of the current study, we cannot determine causality. Longitudinal or experimental research designs are needed to clarify these causal pathways.

#### Sampling bias

A key limitation relates to the recruitment method. The sample was drawn from environmental groups on social media, which likely attracted participants already highly engaged and environmentally aware. This self-selection bias may inflate the observed relationships between ecological anxiety and pro-environmental behavior and limits generalizability. Future studies should strive for more representative and diverse sampling, including individuals not affiliated with environmental organizations.

#### Individual differences

Another important avenue for future research concerns individual difference variables that may moderate the relationship between ecological anxiety and environmental action. Traits such as conscientiousness, environmental identity, and social responsibility may influence how anxiety translates into behavior. Similarly, external motivators like peer influence or political orientation could shape responses. Identifying such moderators or mediators could offer a more nuanced understanding of why ecological anxiety leads to action in some individuals but not in others.

#### Additional considerations

The correlation between age and ecological anxiety—where younger individuals tend to report higher anxiety, while older individuals engage more in personal pro-environmental behaviors—warrants further investigation. This trend may reflect differences in life experience, available resources, or perceived agency. Future research should explore how different age groups experience and respond to ecological anxiety, and how policies can be tailored to their specific psychological and behavioral profiles.

Finally, exploring the underlying psychological mechanisms—such as a sense of control, agency, or community belonging—that link ecological anxiety to environmental behaviors may reveal how these actions serve to fulfill psychological needs. Understanding these mechanisms could help develop strategies to transform ecological anxiety into constructive environmental engagement across diverse populations.

## Data Availability

The raw data supporting the conclusions of this article will be made available by the authors, without undue reservation.

## References

[ref1] Ágoston C. Csaba B. Nagy B. Kőváry Z. Dúll A. Rácz J.. (2022a). Identifying types of eco-anxiety, eco-guilt, eco-grief, and eco-coping in a climate-sensitive population: a qualitative study. Int. J. Environ. Res. Public Health 19:2461. doi: 10.3390/ijerph19042461, PMID: 35206648 PMC8875433

[ref2] Ágoston C. Urbán R. Nagy B. Csaba B. Kőváry Z. Kovács K.. (2022b). The psychological consequences of the ecological crisis: three new questionnaires to assess eco-anxiety, eco-guilt, and ecological grief. Clim. Risk Manag. 37:100441. doi: 10.1016/j.crm.2022.100441

[ref3] Baudon P. Jachens L. (2021). A scoping review of interventions for the treatment of eco-anxiety. Int. J. Environ. Res. Public Health 18:9636. doi: 10.3390/ijerph18189636, PMID: 34574564 PMC8464837

[ref4] Bonneux I. Van Praag L. (2024). ‘Millions of climate refugees are coming!’ A critical media discourse analysis on climate change-induced migrants in Flemish press’. *Environmental*. Sociology 10, 486–498. doi: 10.1080/23251042.2024.2369738, PMID: 40640861

[ref5] Brick C. Lewis G. J. (2016). Unearthing the “green” personality: Core traits predict environmentally friendly behavior. Environ. Behav. 48, 635–658. doi: 10.1177/0013916514554695

[ref6] Brophy H. Olson J. Paul P. (2023). Eco-anxiety in youth: an integrative literature review. Int. J. Ment. Health Nurs. 32, 633–661. doi: 10.1111/inm.13099, PMID: 36582129

[ref7] Chapman S. J. (2017). Review of discovering statistics using IBM SPSS statistics. J. Polit. Sci. Educ. 14, 145–147. doi: 10.1080/15512169.2017.1366328, PMID: 40640861

[ref8] Clarke A. T. (2006). Coping with interpersonal stress and psychosocial health among children and adolescents: a Meta-analysis. J. Youth Adolesc. 35, 10–23. doi: 10.1007/s10964-005-9001-x

[ref9] Clayton S. (2003). Environmental identity: A conceptual and an operational definition. Ident. Nat. Environ, 45–65. doi: 10.7551/mitpress/3644.003.0005

[ref10] Clayton S. Karazsia B. T. (2020). Development and validation of a measure of climate change anxiety. J. Environ. Psychol. 69:101434. doi: 10.1016/j.jenvp.2020.101434, PMID: 40727928

[ref11] Coffey Y. Bhullar N. Joanne Durkin M. Islam S. Usher K. (2021). Understanding eco-anxiety: a systematic scoping review of current literature and identified knowledge gaps. J Climate Change Health 3:100047. doi: 10.1016/j.joclim.2021.100047, PMID: 40727928

[ref12] Cohen J. Cohen P. West S. G. Aiken L. S. (2003). Applied multiple regression/correlation analysis for the behavioral sciences. 3rd Edn. New York: Routledge.

[ref13] Coppola I. (2021). Eco-anxiety in “the climate generation”: Is action an antidote? Environmental Studies Electronic Thesis Collection, Available online at: https://scholarworks.uvm.edu/envstheses/71 (Accessed August 04, 2025).

[ref14] Cronbach L. J. (1951). Coefficient alpha and the internal structure of tests. Psychometrika 16, 297–334. doi: 10.1007/BF02310555

[ref15] Dietz T. Stern P. C. Guagnano G. A. (1998). Social structural and social psychological bases of environmental concern. Environ. Behav. 30, 450–471. doi: 10.1177/001391659803000402

[ref9001] Fielding K. S. Hornsey M. J. (2016). A social identity analysis of climate change and environmental attitudes and behaviors: Insights and opportunities. Front psychol. 7:121. 26903924 10.3389/fpsyg.2016.00121 PMC4749709

[ref16] Fritsche I. Masson T. (2021). Collective climate action: when do people turn into collective environmental agents? Curr. Opin. Psychol. 42, 114–119. doi: 10.1016/j.copsyc.2021.05.001, PMID: 34130199

[ref17] Gifford E. Gifford R. (2016). The largely unacknowledged impact of climate change on mental health. Bull. At. Sci. 72, 292–297. doi: 10.1080/00963402.2016.1216505

[ref19] Innocenti M. Santarelli G. Lombardi G. S. Ciabini L. Zjalic D. Di Russo M.. (2023). How can climate change anxiety induce both pro-environmental Behaviours and eco-paralysis? The mediating role of general self-efficacy. Int. J. Environ. Res. Public Health 20:3085. doi: 10.3390/ijerph20043085, PMID: 36833780 PMC9960236

[ref20] Jain N. Jain P. (2022). Eco-anxiety and environmental concern as predictors of eco-activism. IOP Conference Series 1084:012007. doi: 10.1088/1755-1315/1084/1/012007, PMID: 38865952

[ref21] Kim K. Jhang J. Song S. Shin H. Song S. (2024). Goal proximity effect on collective action: the mediating role of perceived behavioral impact and collective outcome expectancy. J. Bus. Res. 178:114642. doi: 10.1016/j.jbusres.2024.114642, PMID: 40727928

[ref22] Lee Kathryn. (2023). Eco-anxiety in undergraduates: An exploration of Western Washington University students. Beliefs of and Personal Experiences with Climate Change and Ecological Crisis’. *WWU Honors College Senior Projects*, July. Available online at: https://cedar.wwu.edu/wwu_honors/749

[ref23] Loll L. Schmatz N. Von Lonski L. Cremer L. D. Richter M. H. (2023). ‘The influence of climate crisis-related media reporting on the eco-anxiety of individuals’. *Interdisciplinary journal of environmental and science*. Education 19:e 2306. doi: 10.29333/ijese/13044

[ref24] Myers T. A. Nisbet M. C. Maibach E. W. Leiserowitz A. A. (2012). A public health frame arouses hopeful emotions about climate change: a letter. Clim. Chang. 113, 1105–1112. doi: 10.1007/s10584-012-0513-6, PMID: 40728660

[ref25] Nairn K. (2019). Learning from young people engaged in climate activism: the potential of collectivizing despair and Hope. Young 27, 435–450. doi: 10.1177/1103308818817603

[ref26] Ojala M. (2013). Coping with climate change among adolescents: implications for subjective well-being and environmental engagement. Sustainability 5, 2191–2209. doi: 10.3390/su5052191

[ref27] Ojala M. (2023). How do children, adolescents, and young adults relate to climate change? Implications for Developmental Psychology. Eur. J. Dev. Psychol. 20, 929–943. doi: 10.1080/17405629.2022.2108396

[ref28] Ojala M. Cunsolo A. Ogunbode C. A. Middleton J. (2021). Anxiety, worry, and grief in a time of environmental and climate crisis: a narrative review. Annu. Rev. Environ. Resour. 46, 35–58. doi: 10.1146/annurev-environ-012220-022716

[ref29] Pihkala P. (2020a). Anxiety and the ecological crisis: an analysis of eco-anxiety and climate anxiety. Sustainability 12:7836. doi: 10.3390/su12197836

[ref30] Pihkala P. (2020b). Eco-anxiety and environmental education. Sustainability 12:10149. doi: 10.3390/su122310149

[ref31] Ripple W. J. Wolf C. Newsome T. M. Barnard P. Moomaw W. R. (2020). World scientists’ warning of a climate emergency. Bio Science 70, 8–100.258 Scientist Signatories From 153 Countries (List In Supplemental File S1) 11. Available at: https://www.jstor.org/stable/26891410

[ref32] Schäfer T. Schwarz M. A. (2019). The meaningfulness of effect sizes in psychological research: differences between sub-disciplines and the impact of potential biases. Front. Psychol. 10:813. doi: 10.3389/fpsyg.2019.00813, PMID: 31031679 PMC6470248

[ref33] Schwartz S. E. O. Benoit L. Clayton S. Parnes M. K. F. Swenson L. Lowe S. R. (2023). Climate change anxiety and mental health: environmental activism as buffer. Curr. Psychol. 42, 16708–16721. doi: 10.1007/s12144-022-02735-6, PMID: 35250241 PMC8883014

[ref34] Sguin C. Pelletier L. G. Hunsley J. (1998). Toward a model of environmental activism. Environ. Behav. 30, 628–652. doi: 10.1177/001391659803000503

[ref35] Shabat M. Shafir R. Sheppes G. (2021). Flexible emotion regulatory selection when coping with COVID-19-related threats during quarantine. Sci. Rep. 11:21468. doi: 10.1038/s41598-021-00716-6, PMID: 34728671 PMC8563799

[ref36] Stanley S. K. Hogg T. L. Leviston Z. Walker I. (2021). From anger to action: differential impacts of eco-anxiety, eco-depression, and eco-anger on climate action and wellbeing. J. Climate Change Health 1:100003. doi: 10.1016/j.joclim.2021.100003, PMID: 40727928

[ref37] Sternberg N. Luria R. Chandhok S. Vickers B. Kross E. Sheppes G. (2020). When facebook and finals collide-procrastinatory social media usage predicts enhanced anxiety☆. Comput. Hum. Behav. 109:106358. doi: 10.1016/j.chb.2020.106358

[ref38] Venhoeven L. Bolderdijk J. Steg L. (2013). Explaining the paradox: how pro-environmental behaviour can both thwart and Foster well-being. Sustainability 5, 1372–1386. doi: 10.3390/su5041372

[ref39] Wright R. T. (1970). Responsibility for the ecological crisis. Bio Science 20, 851–853. doi: 10.2307/1295493, PMID: 39964225

