# Supplementary material for: Examining the relationship between ecological anxiety and pro-environmental behavior: personal and collective actions
Source: Front Psychol. 2025 Aug 13;16:1505564. doi: 10.3389/fpsyg.2025.1505564 (PMC12382445; doi:10.3389/fpsyg.2025.1505564)
Supplement: Supplementary file 1 [file Supplementary_file_1.docx]

**Examining the Relationship Between Ecological Anxiety and Pro-Environmental Behavior: Personal and Collective Actions**

**Appendix 1 - Personal Pro-environmental behavior.**

Based on Brick and Lewis (2016)

**Rate from 1 (never) to 5 (always)**

1. When you visit the grocery store, how often do you use reusable bags?
2. How often do you walk, ride a bike, use a carpool or take public transportation instead of driving a vehicle by yourself?
3. How often do you go on personal (non-business) air travel?
4. How often do compost your household food garbage?
5. How often do you eat meat (beef, turkey, chicken) and fish?
6. How often do you eat animal products other than meat and fish, such as milk, cheese, eggs or yogurt? [reverse]
7. How often do you eat organic food?
8. How often do you purchase local food (produced in Israel)?
9. How often do you eat from a home vegetable garden (during the growing season)?
10. How often do you turn off your personal electronics or put them into low power mode when the device is not in use?
11. When you buy light bulbs, how often do you buy compact fluorescent (CFL) or high efficiency LEDs?
12. How often do you act to conserve water, when showering, cleaning clothes, dishes, watering plants or other uses?
13. How often do you recycle water, collected from rain, shower or other uses?
14. When you are in PUBLIC, how often do you sort the trash into the recycling?
15. When you are in PRIVATE, how often do you sort the trash into the recycling?

**Appendix 2 - Collective pro environmental behavior.**

Adopted from SGuin, Pelletier, and Hunsley (1998)

**Rate from 1 (never) to 5 (always)**

1. How often do you participate in events organized by environmental groups?
2. How often do you financially support a group that promotes environmental agendas?
3. When you invest money, how often do you check that the money is invested in green stocks/indices?
4. How often do you distribute a petition demanding to improve the government's environmental policy?
5. How often do you participate in a demonstration against the current environmental conditions?
6. In elections, how often do you vote for a government that offers environmentally conscious policies?
7. How often do you write a letter to firms that produce products that are harmful to the environment?

**Appendix 3 – Ecological anxiety**

**Rate from 1 (never) to 5 (always)**

People have many different thoughts, feelings, and reactions regarding climate change and the ecological crisis. Please indicate how strongly you agree or disagree with the following statements (1 Strongly disagree, 2 Somewhat disagree, 3 Somewhat agree, 4 Agree, 5 Strongly agree). (Ágoston, Urbán, et al. 2022)

1. It really upsets me to see how animals are suffering because of environmental pollution.
2. I worry about the next generation because they will be drastically affected by climate change.
3. I am so anxious about climate change that I cry.
4. It makes me angry that many people fail to do even the most basic things to protect the environment.
5. I have unusual tension in my muscles since I've become more aware of climate change.
6. I feel sorry for those whose health is already negatively affected by climate change.
7. I am terrified by how many things have changed in just a few years because of climate change.
8. My loved ones become irritated because I talk about my climate change concerns too often.
9. I am worried about the increasing number of natural disasters caused by climate change.
10. Thoughts of climate change often distract me from my current tasks.
11. It makes me sick to think about how much certain countries are polluting the environment, and there is nothing I can do about it.
12. It scares me that the weather is becoming more and more unpredictable because of climate change.
13. I am so anxious about climate change that it affects my performance at school/work.
14. It is frustrating that we elect decision makers who do not seriously consider the work of climate scientists/experts.
15. I feel uneasy when I think about the consequences of climate change.
16. People look at me in a strange way, because I am so passionate about environmental action.
17. I find it terrifying that the seasons have changed a lot in a short time.
18. I worry that every decision I make will result in something harmful to the environment.
19. It makes me angry that our environmentally damaging behaviors increase the suffering of people who live in areas that are more impacted by climate change.
20. I have a very negative perspective on the future of the planet because of climate change.
21. I am constantly on alert because there could be a climate change related disaster at any time.
22. I sleep poorly because I keep thinking about climate change.

**Appendix 4 - Power Analysis**

Based on a previous studies ( Schäfer et al, 2019), that also involved two distinct questionnaires, we set a conservative estimated effect size of Pearson's r = 0.23 in a formal power analysis (using G power 3.1.9.4), applying the conventional high power of 0.95 and an alpha of 0.05 (notably, the observed effect sizes Pearson's r = 0.23 and Pearson's r  = 0.81 reported below confirmed and exceeded the expected effect size). The formal power analysis pointed to a sample size of 191 participants. Thus, we aimed to recruit 230 participants, taking into account 15-20% exclusion rates in online studies (Sternberg, N. et al. 2020, Shabat, M et al, 2021). The final sample consisted of 226 participants.

**References**

Ágoston, Csilla, Róbert Urbán, Bence Nagy, Benedek Csaba, Zoltán Kőváry, Kristóf Kovács, Attila Varga, et al. 2022. ‘The Psychological Consequences of the Ecological Crisis: Three New Questionnaires to Assess Eco-Anxiety, Eco-Guilt, and Ecological Grief’. *Climate Risk Management* 37:100441. <https://doi.org/10.1016/j.crm.2022.100441>

Brick, Cameron, and Gary J. Lewis. 2016. ‘Unearthing the “Green” Personality: Core Traits Predict Environmentally Friendly Behavior’. *Environment and Behavior* 48 (5): 635–58. <https://doi.org/10.1177/0013916514554695>.

Schäfer, Thomas, and Marcus A. Schwarz. "The meaningfulness of effect sizes in psychological research: Differences between sub-disciplines and the impact of potential biases." *Frontiers in psychology* 10 (2019): 813.

SGuin, Chantal, Luc G. Pelletier, and John Hunsley. 1998. ‘Toward a Model of Environmental Activism’. *Environment and Behavior* 30 (5): 628–52. <https://doi.org/10.1177/001391659803000503>.

Shabat, Maya, Roni Shafir, and Gal Sheppes. "Flexible emotion regulatory selection when coping with COVID-19-related threats during quarantine." Scientific Reports 11, no. 1 (2021): 21468.

Stanley, Samantha K., Teaghan L. Hogg, Zoe Leviston, and Iain Walker. 2021. ‘From Anger to Action: Differential Impacts of Eco-Anxiety, Eco-Depression, and Eco-Anger on Climate Action and Wellbeing’. *The Journal of Climate Change and Health* 1 (March):100003. <https://doi.org/10.1016/j.joclim.2021.100003>.
